# Supplementary material for: Characteristics of putative keystones in the healthy adult human gut microbiota as determined by correlation network analysis
Source: Front Microbiol. 2024 Nov 20;15:1454634. doi: 10.3389/fmicb.2024.1454634 (PMC11614764; doi:10.3389/fmicb.2024.1454634)
Supplement: Supplementary file 4 [file Data_Sheet_1.pdf]

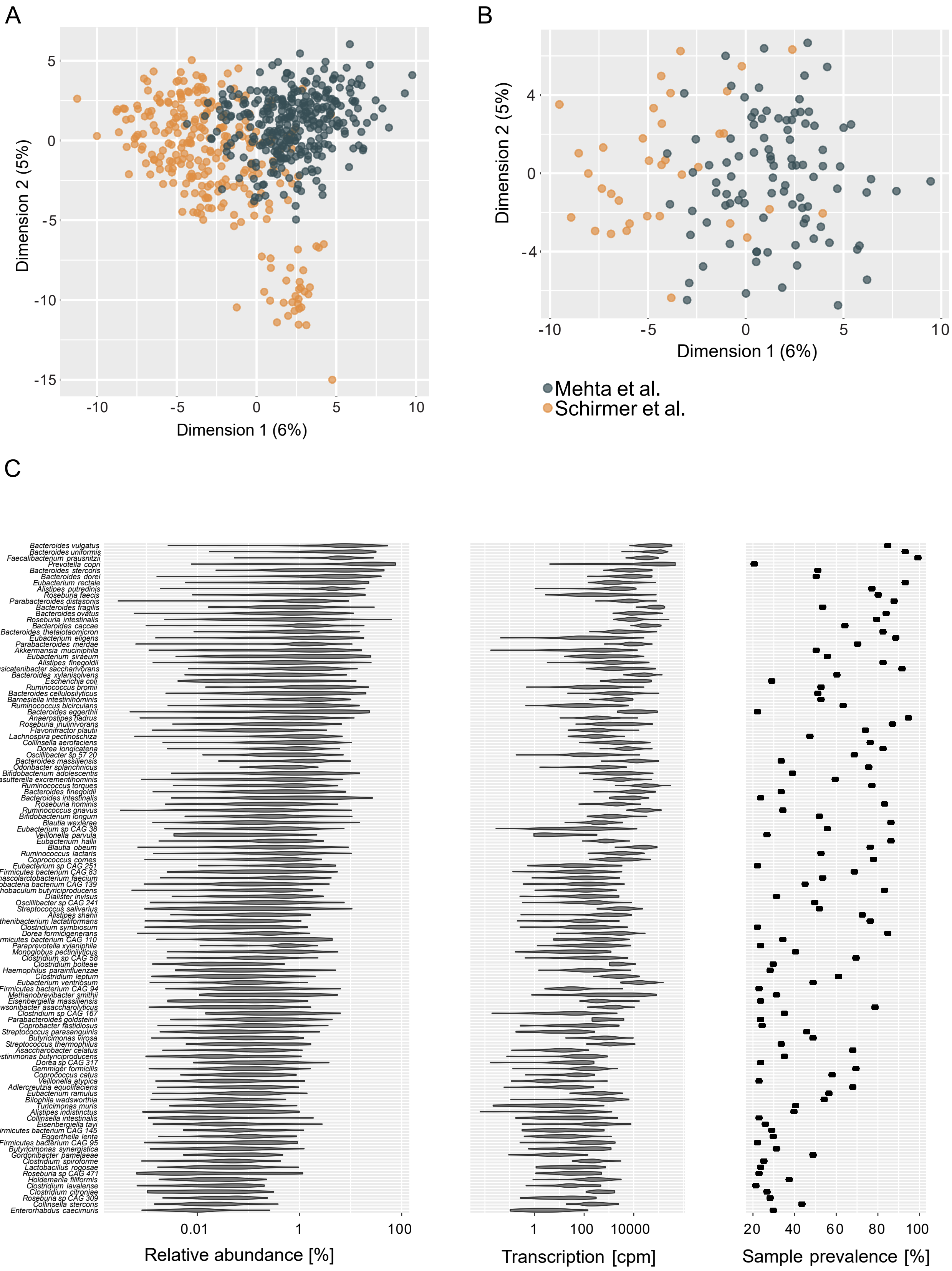
A
B
C
Dimension 1 (6%)
Dimension 2 (5%)
Mehta et al.
Schirmer et al.
Relative abundance [%]
Transcription [cpm]
Sample prevalence [%]

Figure S1 Principal coordinate analysis of (**A**) all processed samples and (**B**) samples used for further analysis (one randomly selected sample per participant). Depicted is the robust Aitchison distance of estimated total reads mapped to species reference genomes. Colors indicate the study that published the raw data. Panel **C** shows relative abundance [%], transcription [cpm - copies per million] and sample prevalence [%] of species present in at least 20% of analyzed samples. Species are ordered by mean relative abundance.

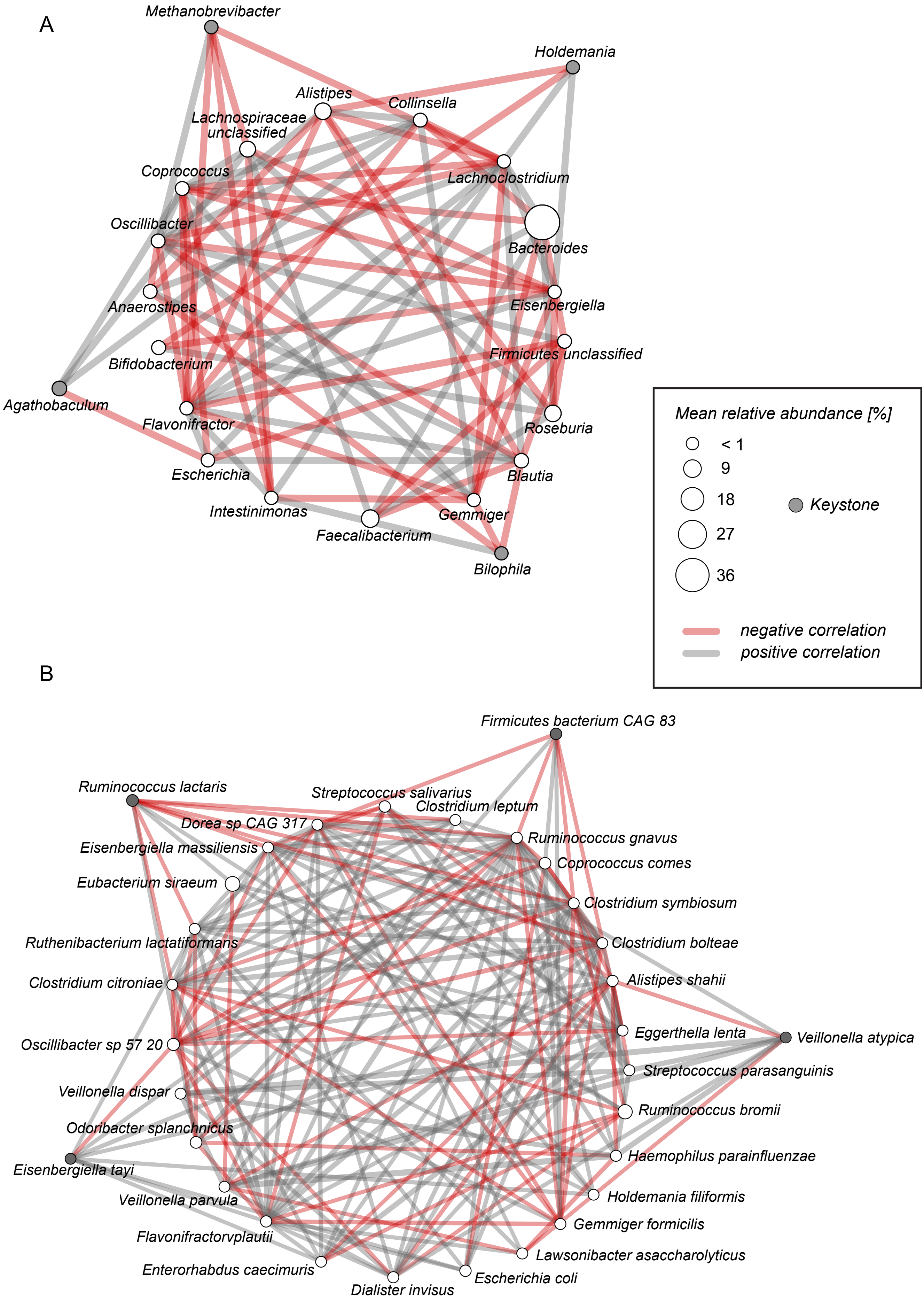
A
Methanobrevibacter
Holdemania
Alistipes
Collinsella
Lachnospiraceae unclassified
Coprococcus
Lachnoclostridium
Oscillibacter
Bacteroides
Anaerostipes
Eisenbergiella
Bifidobacterium
Firmicutes unclassified
Agathobaculum
Flavonifractor
Roseburia
Escherichia
Blautia
Intestinimonas
Gemmiger
Faecalibacterium
Bilophila
Mean relative abundance [%]
< 1
9
18
27
36
Keystone
negative correlation
positive correlation
B
Firmicutes bacterium CAG 83
Ruminococcus lactaris
Streptococcus salivarius
Clostridium leptum
Dorea sp CAG 317
Ruminococcus gnavus
Eisenbergiella massiliensis
Coprococcus comes
Eubacterium siraeum
Clostridium symbiosum
Ruthenibacterium lactatiformans
Clostridium bolteae
Clostridium citroniae
Alistipes shahii
Eggerthella lenta
Veillonella atypica
Oscillibacter sp 57 20
Streptococcus parasanguinis
Veillonella dispar
Ruminococcus bromii
Odoribacter splanchnicus
Haemophilus parainfluenzae
Eisenbergiella tayi
Holdemania filiformis
Veillonella parvula
Gemmiger formicilis
Flavonifractorvplautii
Lawsonibacter asaccharolyticus
Enterorhabdus caecimuris
Escherichia coli
Dialister invisus

Figure S2 Correlation networks showing keystone taxa and their first neighbours (taxa directly correlated to a keystone taxon) on (**A**) genus and (**B**) species level. Network nodes in grey (white) indicate keystone taxa (non-keystone taxa), network edges connecting the circles indicate positive (grey) and negative (red) correlations between taxa. Node size indicates mean relative abundance of a given taxon across analyzed samples. Exact correlation coefficients can be found in Table S2.

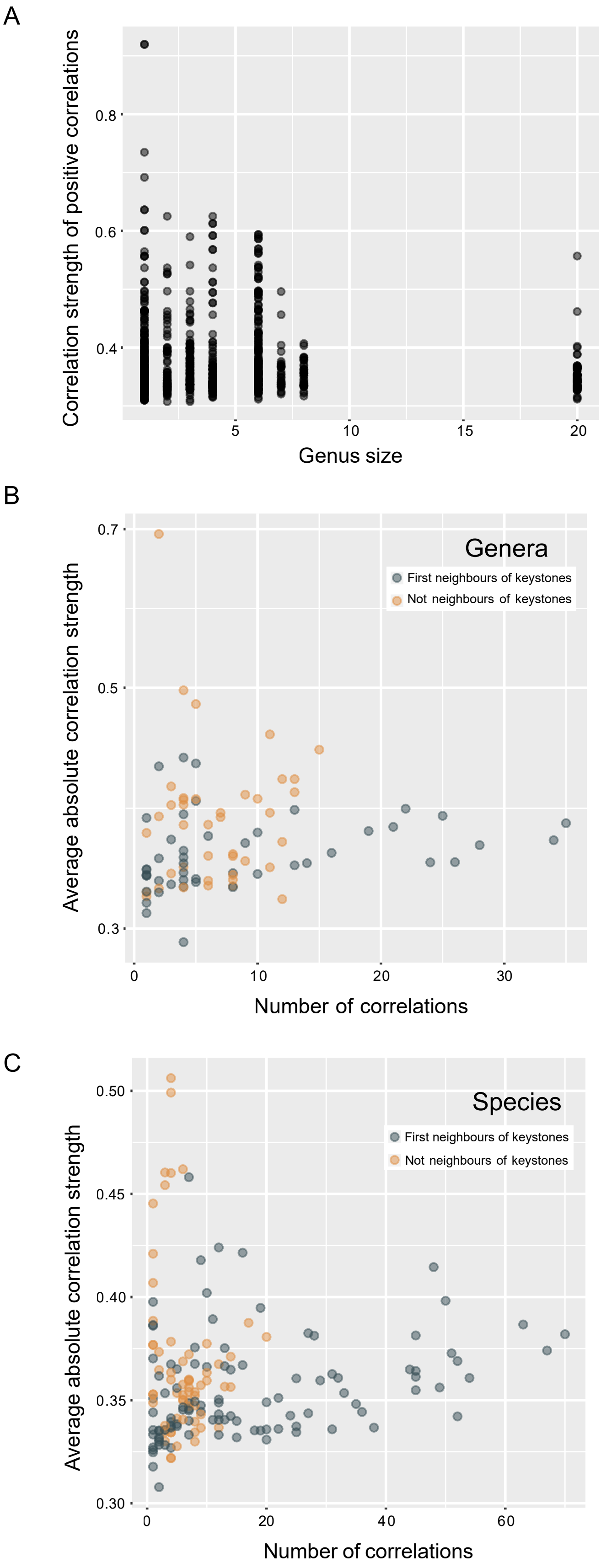
A
Correlation strength of positive correlations
0.8
0.6
0.4
5
10
15
20
Genus size
B
Genera
First neighbours of keystones
Not neighbours of keystones
Average absolute correlation strength
0.7
0.5
0.3
0
10
20
30
Number of correlations
C
Species
First neighbours of keystones
Not neighbours of keystones
Average absolute correlation strength
0.50
0.45
0.40
0.35
0.30
0
20
40
60
Number of correlations

Figure S3 Distributions of network correlations in (**A,C**) the species-level network and (**B**) the genus-level network. Panel **A** shows the within-genus correlation strength of positive correlations as a factor of genus size. Panels **B** and **C** depict the average absolute correlation strength compared to the total number of correlations in (**B**) the genus-level network and (**C**) the species-level network. Gray circles indicate taxa that are first neighbors of (= directly correlated with) a keystone taxon, orange circles indicate taxa that are not first neighbors of any keystone taxa.

A Genera

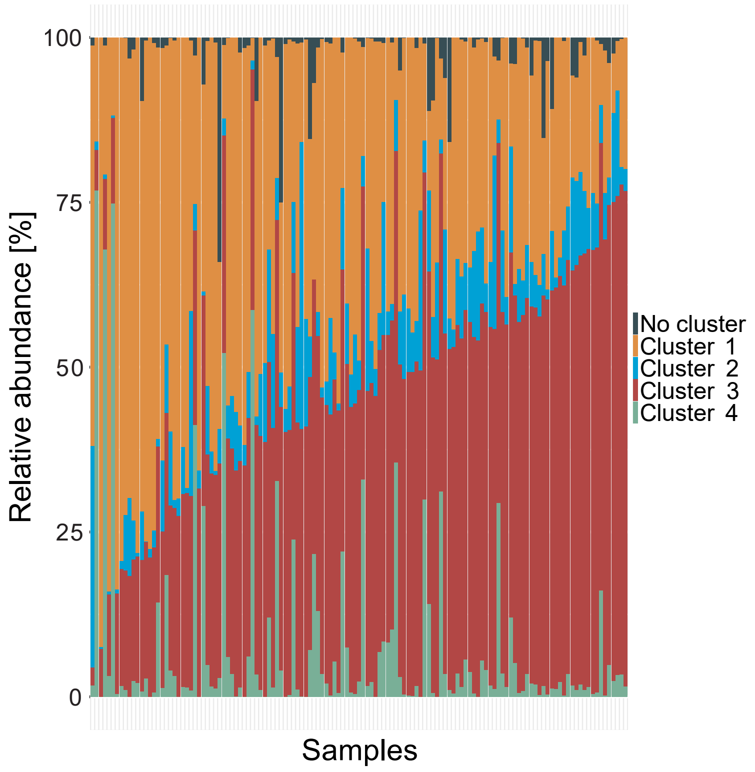

B Species

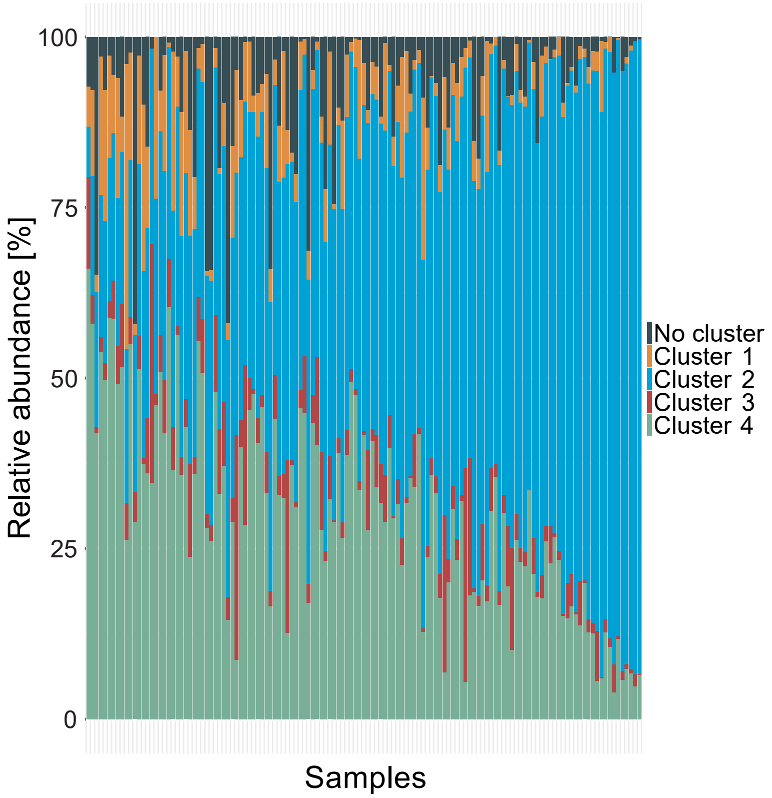

Figure S4 Relative abundance [%] of co-occurrence network clusters of (**A**) genera and (**B**) species. Colors indicate the respective cluster that taxa were grouped into (Fig 5), gray indicates taxa not grouped into any cluster. Samples were ordered based on a k-means clustering algorithm. (**A**) Genus network cluster 2 includes the keystone genera *Bilophila* and *Holdemania* and cluster 3 includes the keystone genera *Agathobaculum* and *Methanobrevibacter.* (**B**) Species network cluster 1 includes the keystone species *Veillonella atypica*, cluster 2 includes *Ruminococcus lactaris*, cluster 3 includes *Eisenbergiella tayi* and cluster 4 includes *Firmicutes bacterium* CAG 83 (unclassified *Bacillota*).

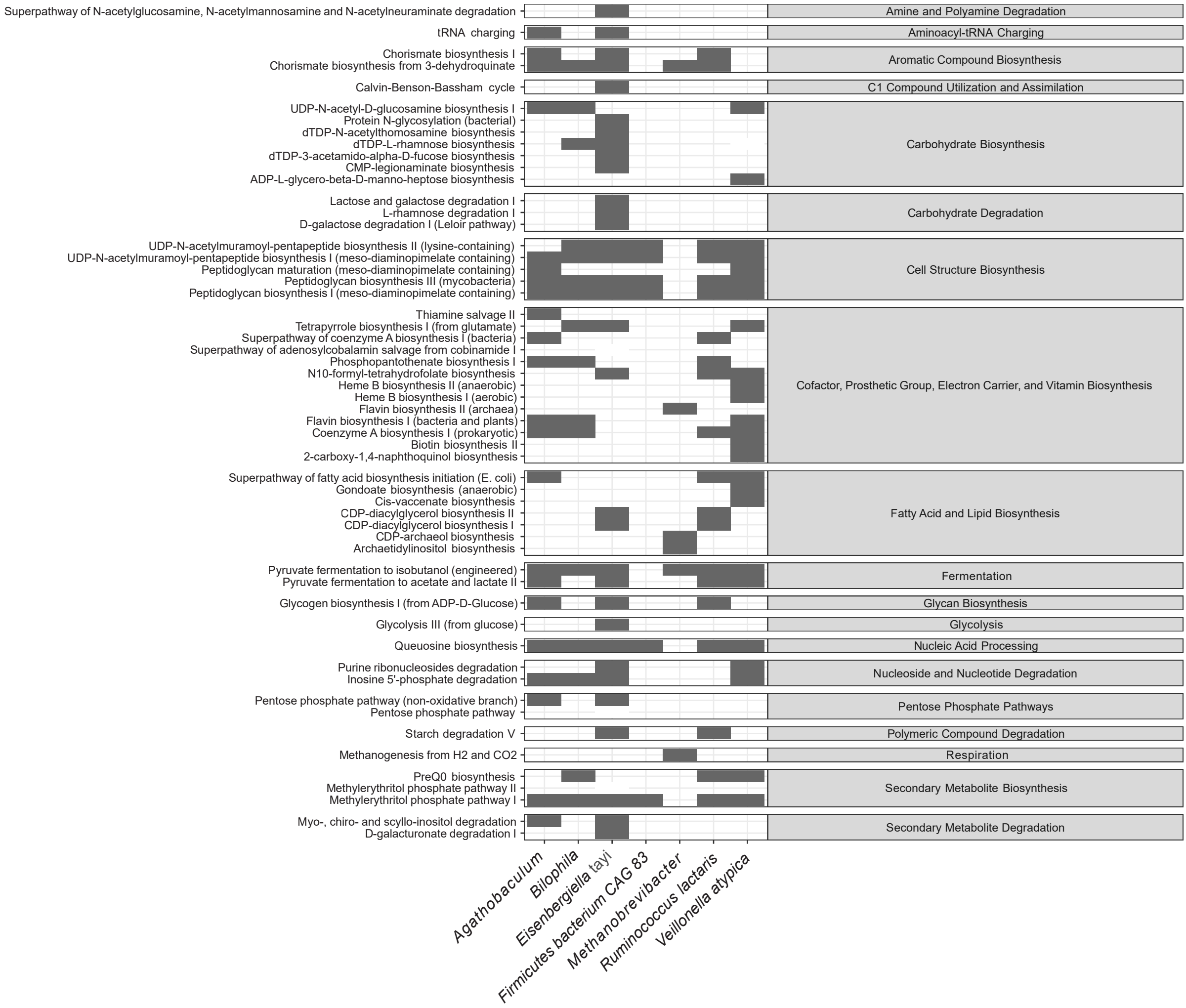

Superpathway of N-acetylglucosamine, N-acetylmannosamine and N-acetylneuraminate degradation
tRNA charging
Chorismate biosynthesis I
Chorismate biosynthesis from 3-dehydroquinate
Calvin-Benson-Bassham cycle
UDP-N-acetyl-D-glucosamine biosynthesis I
Protein N-glycosylation (bacterial)
dTDP-N-acetylthomosamine biosynthesis
dTDP-L-rhamnose biosynthesis
dTDP-3-acetamido-alpha-D-fucose biosynthesis
CMP-legionaminate biosynthesis
ADP-L-glycero-beta-D-manno-heptose biosynthesis
Lactose and galactose degradation I
L-rhamnose degradation I
D-galactose degradation I (Leloir pathway)
UDP-N-acetylmuramoyl-pentapeptide biosynthesis II (lysine-containing)
UDP-N-acetylmuramoyl-pentapeptide biosynthesis I (meso-diaminopimelate containing)
Peptidoglycan maturation (meso-diaminopimelate containing)
Peptidoglycan biosynthesis III (mycobacteria)
Peptidoglycan biosynthesis I (meso-diaminopimelate containing)
Thiamine salvage II
Tetrapyrrole biosynthesis I (from glutamate)
Superpathway of coenzyme A biosynthesis I (bacteria)
Superpathway of adenosylcobalamin salvage from cobinamide I
Phosphopantothenate biosynthesis I
N10-formyl-tetrahydrofolate biosynthesis
Heme B biosynthesis II (anaerobic)
Heme B biosynthesis I (aerobic)
Flavin biosynthesis II (archaea)
Flavin biosynthesis I (bacteria and plants)
Coenzyme A biosynthesis I (prokaryotic)
Biotin biosynthesis II
2-carboxy-1,4-naphthoquinol biosynthesis
Superpathway of fatty acid biosynthesis initiation (E. coli)
Gondoate biosynthesis (anaerobic)
Cis-vaccenate biosynthesis
CDP-diacylglycerol biosynthesis II
CDP-diacylglycerol biosynthesis I
CDP-archaeol biosynthesis
Archaetidylinositol biosynthesis
Pyruvate fermentation to isobutanol (engineered)
Pyruvate fermentation to acetate and lactate II
Glycogen biosynthesis I (from ADP-D-Glucose)
Glycolysis III (from glucose)
Queuosine biosynthesis
Purine ribonucleosides degradation
Inosine 5'-phosphate degradation
Pentose phosphate pathway (non-oxidative branch)
Pentose phosphate pathway
Starch degradation V
Methanogenesis from H2 and CO2
PreQ0 biosynthesis
Methylerythritol phosphate pathway II
Methylerythritol phosphate pathway I
Myo-, chiro- and scyllo-inositol degradation
D-galacturonate degradation I
Amine and Polyamine Degradation
Aminoacyl-tRNA Charging
Aromatic Compound Biosynthesis
C1 Compound Utilization and Assimilation
Carbohydrate Biosynthesis
Carbohydrate Degradation
Cell Structure Biosynthesis
Cofactor, Prosthetic Group, Electron Carrier, and Vitamin Biosynthesis
Fatty Acid and Lipid Biosynthesis
Fermentation
Glycan Biosynthesis
Glycolysis
Nucleic Acid Processing
Nucleoside and Nucleotide Degradation
Pentose Phosphate Pathways
Polymeric Compound Degradation
Respiration
Secondary Metabolite Biosynthesis
Secondary Metabolite Degradation
Agathobaculum
Bilophila
Eisenbergiella tayi
Firmicutes bacterium CAG 83
Methanobrevibacter
Ruminococcus lactaris
Veillonella atypica

Figure S5 Presence of metabolic pathways in identified correlation network keystone taxa. Gray color indicates that a pathway was found to be present in the metagenome reads mapped to the respective taxon.

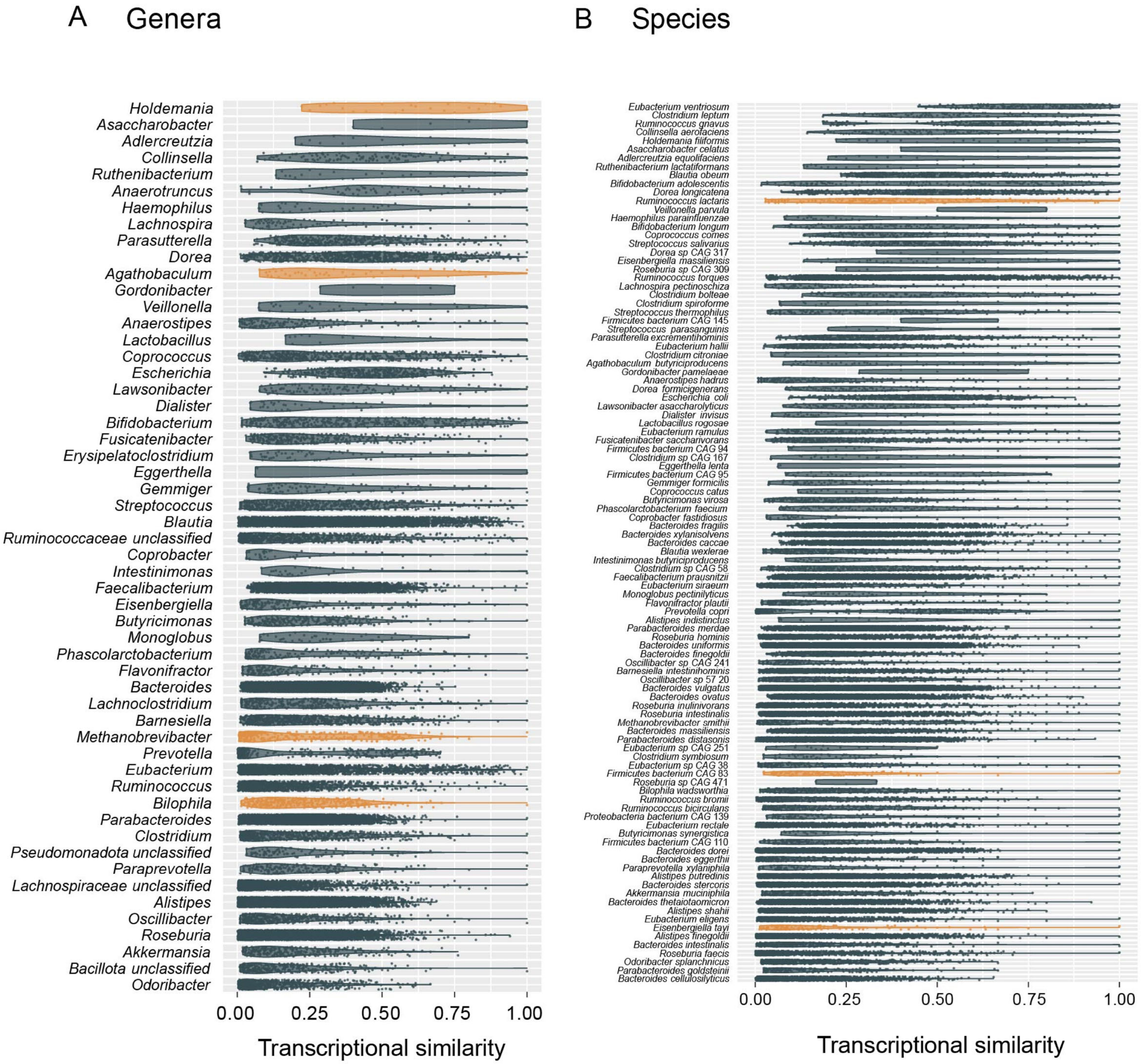

A
Genera
Holdemania
Asaccharobacter
Adlercreutzia
Collinsella
Ruthenibacterium
Anaerotruncus
Haemophilus
Lachnospira
Parasutterella
Dorea
Agathobaculum
Gordonibacter
Veillonella
Anaerostipes
Lactobacillus
Coprococcus
Escherichia
Lawsonibacter
Dialister
Bifidobacterium
Fusicatenibacter
Erysipelatoclostridium
Eggerthella
Gemmiger
Streptococcus
Blautia
Ruminococcaceae unclassified
Coprobacter
Intestinimonas
Faecalibacterium
Eisenbergiella
Butyricimonas
Monoglobus
Phascolarctobacterium
Flavonifractor
Bacteroides
Lachnoclostridium
Barnesiella
Methanobrevibacter
Prevotella
Eubacterium
Ruminococcus
Bilophila
Parabacteroides
Clostridium
Pseudomonadota unclassified
Paraprevotella
Lachnospiraceae unclassified
Alistipes
Oscillibacter
Roseburia
Akkermansia
Bacillota unclassified
Odoribacter
0.00
0.25
0.50
0.75
1.00
Transcriptional similarity
B
Species
Eubacterium ventriosum
Clostridium leptum
Ruminococcus gnavus
Collinsella aerofaciens
Holdemania filiformis
Asaccharobacter celatus
Adlercreutzia equolifaciens
Ruthenibacterium lactatiformans
Blautia obeum
Bifidobacterium adolescentis
Dorea longicatena
Ruminococcus lactaris
Veillonella parvula
Haemophilus parainfluenzae
Bifidobacterium longum
Coprococcus comes
Streptococcus salivarius
Dorea sp CAG 317
Eisenbergiella massiliensis
Roseburia sp CAG 309
Ruminococcus torques
Lachnospira pectinoschiza
Clostridium bolteae
Clostridium spiroforme
Streptococcus thermophilus
Firmicutes bacterium CAG 145
Streptococcus parasanguinis
Parasutterella excrementihominis
Eubacterium hallii
Clostridium citroniae
Agathobaculum butyriciproducens
Gordonibacter pamelaeae
Anaerostipes hadrus
Dorea formicigenerans
Escherichia coli
Lawsonibacter asaccharolyticus
Dialister invisus
Lactobacillus rogosae
Eubacterium ramulus
Fusicatenibacter saccharivorans
Firmicutes bacterium CAG 94
Clostridium sp CAG 167
Eggerthella lenta
Firmicutes bacterium CAG 95
Gemmiger formicilis
Coprococcus catus
Butyricimonas virosa
Phascolarctobacterium faecium
Coprobacter fastidiosus
Bacteroides fragilis
Bacteroides xylanisolvens
Bacteroides caccae
Blautia wexlerae
Intestinimonas butyriciproducens
Clostridium sp CAG 58
Faecalibacterium prausnitzii
Eubacterium siraeum
Monoglobus pectinilyticus
Flavonifractor plautii
Prevotella copri
Alistipes indistinctus
Parabacteroides merdae
Roseburia hominis
Bacteroides uniformis
Bacteroides finegoldii
Oscillibacter sp CAG 241
Barnesiella intestinihominis
Oscillibacter sp 57 20
Bacteroides vulgatus
Bacteroides ovatus
Roseburia inulinivorans
Roseburia intestinalis
Methanobrevibacter smithii
Bacteroides massiliensis
Parabacteroides distasonis
Eubacterium sp CAG 251
Clostridium symbiosum
Eubacterium sp CAG 38
Firmicutes bacterium CAG 83
Roseburia sp CAG 471
Bilophila wadsworthia
Ruminococcus bromii
Ruminococcus bicirculans
Proteobacteria bacterium CAG 139
Eubacterium rectale
Butyricimonas synergistica
Firmicutes bacterium CAG 110
Bacteroides dorei
Bacteroides eggerthii
Paraprevotella xylaniphila
Alistipes putredinis
Bacteroides stercoris
Akkermansia muciniphila
Bacteroides thetaiotaomicron
Alistipes shahii
Eubacterium eligens
Eisenbergiella tayi
Alistipes finegoldii
Bacteroides intestinalis
Roseburia faecis
Odoribacter splanchnicus
Parabacteroides goldsteinii
Bacteroides cellulosilyticus
0.00
0.25
0.50
0.75
1.00
Transcriptional similarity

Figure S6 Transcriptional similarity of (**A**) genera and (**B**) species, with 1 indicating identical transcriptional profiles and 0 indicating no overlap. Transcriptional similarity was calculated as pairwise Sorenson distance from the presence and absence of gene families in the metatranscriptomes. Orange color marks the identified keystone taxa. Taxa are ordered by mean Sorenson distance.
